# Does Prophylactic Oral Zinc Reduce the Risk of Contracting COVID-19?

**DOI:** 10.7759/cureus.30881

**Published:** 2022-10-30

**Authors:** Sean D Adrean, Kenneth Schmitt, Caleb Ng, Ash Pirouz, Hema L Ramkumar, Scott Grant

**Affiliations:** 1 Ophthalmology/Retina, Retina Consultants of Orange County, Fullerton, USA; 2 Ophthalmology, University of California Irvine, Irvine, USA

**Keywords:** public health, macular degeneration, areds vitamins, ophthalmology, zinc supplementation, covid-19

## Abstract

Objective

In this study, we aimed to investigate whether zinc provided in Age-Related Eye Disease Study 2 (AREDS2) vitamins is associated with a decreased risk of contracting coronavirus disease 2019 (COVID-19).

Materials and methods

We conducted a retrospective observational cohort study involving patients at a retina-only practice who were provided a questionnaire at each visit to assess whether they were symptomatic of or had contracted COVID-19. Those who answered yes to testing positive for COVID-19 were retrospectively analyzed and categorized based on their AREDS2 vitamin use, and a Pearson’s chi-squared test was performed. Demographic data and past ocular history were also analyzed.

Results

A total of 8,426 unique patients, including 2,111 with a diagnosis of age-related macular degeneration (AMD), were seen from April 1, 2020, to April 9, 2021. A total of 110 patients (1.3%) reported contracting COVID-19 and had positive COVID-19 tests. The average age of those who had contracted COVID-19 in this study was 68.3 years; 51.8% were male, 30.1% had AMD, 28.2% had diabetic retinopathy, 24.5% had surgical retinal disease, 11.8% had retinal vascular disease, and 4.5% had other disease states. Of the COVID-19-positive patients, 27.3% (30/110) took AREDS2 vitamins, while 72.7% (80/110) patients did not. A chi-squared analysis was performed, which was not statistically significant (p=0.667).

Conclusions

Oral zinc supplementation, in the form of AREDS2 vitamins, is not associated with a protective effect against contracting COVID-19.

## Introduction

Severe acute respiratory syndrome coronavirus 2 (SARS-CoV-2), which causes coronavirus disease 2019 (COVID-19), is a single-stranded, positive-strand RNA virus that belongs to a larger group of human coronaviruses including those that cause Severe Acute Respiratory Syndrome (SARS) and Middle East Respiratory Syndrome (MERS). The highly contagious nature of the virus, rapid infection rate, and continual mutations have led to poor management outcomes with conventional anti-viral treatment [1]. Symptoms of those infected with SARS-CoV-2 (COVID-19) range from being asymptomatic to respiratory failure followed by multiorgan failure and death. The initial therapeutic approach largely consists of patient isolation with supportive medical care until hospitalization is required [2-5]. The availability of safe and highly effective COVID-19 vaccines has increased the possibility of sustainable control of the virus’s spread both in the US and worldwide. The messenger RNA (mRNA) vaccines were initially 95% effective against COVID-19 but were found to be less effective against the Omicron variant. Another anti-viral vaccine that was developed, Janssen viral vector COVID-19 vaccine, was reported to have a 67% efficacy at ≥14 days [6,7]. However, the latest surge in COVID-19 infections has been associated with BA.2.12.1, a sub-variant of Omicron, and the effectiveness of vaccines against the Omicron variant or other new variants is still unclear. Recent studies on this topic indicate that two doses of Moderna or Pfizer/BioNTech mRNA vaccines provide a relatively lower protection against the Omicron variant of COVID-19 and effectivity may wane over time, requiring at least one booster dose, especially in immunocompromised or elderly patients in whom the initial vaccination may not have generated a robust immune response [8]. The effectiveness of vaccines against the Omicron variant is not yet known.

Given the continued need for better treatment options for patients infected with COVID-19, multiple clinical trials are underway to assess the efficacy of certain vitamins and supplements in the treatment of COVID-19. Clinical trials studying the effectiveness of vitamin C, vitamin D, medical cannabis, and zinc are currently in progress (NCT04335084, NCT03944447, NCT04551339). The preventive and therapeutic efficacy of zinc for the treatment of COVID-19 is also under investigation [9]. 

Zinc is an essential bio-metal required for the maintenance and strength of the adaptive and innate immune system [10]. Zinc deficiency has been associated with pathologic conditions such as delayed wound healing and tissue repair, and increased risk of critical illness [11-13]. Furthermore, zinc supplementation has also been shown to inhibit viral replication for the common cold and respiratory syncytial virus infections [14,15]. More importantly, zinc has been reported to inhibit SARS-CoV RNA-dependent RNA polymerase in vitro [16]. While zinc supplementation shows much promise as a therapeutic agent for patients with SARS-CoV-2, little data is available regarding its prophylactic usage against SARS-CoV-2, even though it has been studied as a preventative agent against other viruses [17].

Many patients with age-related macular degeneration (AMD) take Age-Related Eye Disease Study 2 (AREDS2) vitamins, a common long-term, supplemental approach to reduce the risk of progression of macular degeneration, which contains 80 mg of zinc, in the form of zinc oxide [18]. As AREDS2 vitamins have been proven to be safe and are largely accessible, there has been an interest in further studies regarding their usefulness as a prophylactic treatment for those at risk for COVID-19 [19]. We conducted a retrospective analysis to examine whether zinc supplementation in the form of AREDS2 vitamins could prophylactically reduce the risk of contracting COVID-19.

## Materials and methods

We performed a retrospective analysis of data on patients seen at a retina-only private practice in Southern California during the period from April 1, 2020, through April 9, 2021. Local Institutional review board (IRB) approval was obtained (IRB00012874 Retina Consultants of Orange County IRB #1; approval #: 2021-002-RCOC). All data were fully anonymized before they were accessed, and they were collected in accordance with the Health Insurance Portability and Accountability Act (HIPAA). The IRB committee waived the requirement for informed consent. The study adhered to the tenets set forth in the Declaration of Helsinki.

At each visit, at the beginning of the pandemic, patients were required to answer a questionnaire to assess whether they had contracted COVID-19 or possessed symptoms suspicious of infection (Appendix 1). This form was discontinued in April 2021, as vaccinations became more widely available, and the local numbers of positive patients decreased.

This was done to safely care for all patients during the ongoing pandemic. As part of routine intake, the patients are queried about all medications they take, including the specific eye vitamin formulation and other supplements. This information is routinely confirmed by the physician.

Patients who tested positive for COVID-19 and those with suspicious symptoms and later tested positive for COVID-19 were included in the analysis. The electronic medical records of study patients were reviewed. Patients' age, gender, primary retinal pathology, and use of AREDS2 vitamins and other supplements were recorded.

Patients positive for COVID-19 were then categorized based on their usage of AREDS2 vitamins, and a Pearson’s chi-squared test was performed. A p-value less than 0.05 was considered statistically significant. All analyses were performed using R statistical software (R Foundation for Statistical Computing, Vienna, Austria).

## Results

A total of 8,426 unique patients were seen from April 1, 2020, through April 9, 2021. Among those, a total of 110 patients (1.3%) had contracted COVID-19 and had positive COVID-19 tests. One patient died due to COVID-19-related complications (mortality rate: 0.91%). The average age of patients who contracted COVID-19 was 68.3 years (range: 26-93 years) and 51.8% were male. Of those patients who tested positive for COVID-19, 30.1% had AMD, 28.2% had diabetic retinopathy, 24.5% had surgical retinal disease, 11.8% had retinal vascular disease, and 4.5% had other disease states. Of the COVID-19-positive patients, 30 routinely took AREDS2 vitamins, while 80 did not take AREDS2 vitamins. Those who took the AREDS2 vitamins did not differ significantly in age, sex, or past ocular history (Table 1).

**Table 1:** Baseline demographics and clinical characteristics

Variables	Frequency	Percentage	Mean
Gender			
Male	57	51.8%	
Female	53	48.2%	
Age			68.3
History			
Age-related macular degeneration (AMD)	33	30.0%	
Diabetic retinopathy	31	28.2%	
Surgical retinal disease	26	23.6%	
Retinal vascular disease	12	10.9%	
Other disease states	8	7.3%	

A chi-squared analysis was performed (95% CI: −0.066 to 0.111; p=0.667), which was not statistically significant (Table 2).

**Table 2:** 2x2 chi-square contingency table analysis between AREDS2 vitamin supplementation and COVID-19 contagion status AREDS2: Age-Related Eye Disease Study 2; COVID-19: coronavirus disease 2019

	AREDS2 vitamin supplementation	No AREDS2 vitamin supplementation	Total	Statistics
COVID-19-positive	30	80	110	Chi-squared=0.18487
COVID-19-negative	2081	6235	8316	95% CI: -0.66 to 0.111
Total	2111	6315	8426	p=0.6672

## Discussion

As COVID-19 quickly became widespread among the general population, many non-essential businesses were closed. Elective surgeries were delayed and there was concern that the rising number of patients infected with COVID-19 would threaten the healthcare systems worldwide. Many physicians used telemedicine to limit the spread. While this was the case for many specialties, many patients with retinal diseases required timely medical treatments. During the first 10 weeks of the pandemic, only those patients requiring intravitreal injections or laser therapy, for any diagnosis, were cared for, as well as those retinal patients who required urgent or emergent surgical care. Systems were put in place for social distancing and patients were given a form at each visit to see if they had contracted COVID-19. Those patients who had COVID-19 or its symptoms were asked to reschedule their appointment for two weeks. Notably, patients with AMD needed continued anti-VEGF treatment.

We sought to study whether prophylactic zinc could reduce the risk of contracting COVID-19. We hypothesized that if patients took zinc in the form of AREDS2 vitamins and if this led to a lower incidence of COVID-19, then there might be indirect evidence to suggest that zinc supplementation would prophylactically reduce the risk of contracting COVID-19. First, we looked into all COVID-19-positive patients. We classified them by diagnosis and history to see if they took AREDS2 supplementation. In general, patients were asked at least several times a year if they were taking their AREDS vitamins, which consist of 500 mg vitamin C, 400 IU vitamin E, 80 mg zinc, 2 mg copper, 10 mg lutein, and 2 mg zeaxanthin. This regimen has been shown to be effective in slowing the progression of AMD from intermediate to advanced forms by 25% [20].

Of the supplemental components noted within the AREDS 2 vitamin, zinc and vitamin C have become particularly noteworthy topics for consideration for both preventive and therapeutic treatment of COVID-19. Vitamin C is easily obtained in our diet and is in many multivitamins at therapeutic levels, making it more difficult to study. Zinc is known to play an important role in viral immunity and, more notably, is linked with the transmission of coronavirus [16]. Specifically, angiotensin-converting enzyme 2 is a zinc metalloprotease, which is important for the cellular entry of COVID-19 [21]. Additionally, zinc has been shown to inhibit viral RNA-dependent RNA polymerase, specifically against SARS-CoV in vitro [16]. A recent meta-analysis examining zinc for the prevention and treatment of respiratory tract infections (RTIs) found some evidence to suggest that zinc might prevent some symptoms and shorten the duration of the RTIs [22]. Although much has been theoretically reported on the immunological benefits of zinc supplementation, to the best of our knowledge, there has been no study examining the effect of prophylactic zinc on reducing the risk of contracting COVID-19.

Given its association with immune system function and antiviral replication properties, the consideration of zinc as a prophylactic supplement has been previously proposed [10,14-16]. Additionally, zinc is readily accessible to the public and commonly sold at health stores either alone or in combination with other vitamins. When zinc is sold as a supplement alone, it typically contains between 30-50 mg of zinc and when it is in combination with vitamin C, it typically has 30 mg of zinc. Most AREDS2 vitamin formulations sold over the counter have between 40-80 mg of zinc. These characteristics supposedly make zinc an ideal supplement for prophylactic treatment and such attention to zinc has led to studies of zinc supplementation in patients hospitalized with COVID-19.

A recent observational study looking at high doses of daily oral zinc sulfate (440 mg) in hospitalized COVID-19 patients showed no significant difference in the risk of in-hospital mortality [23]. Furthermore, a randomized clinical trial looking at the effects of zinc gluconate (50 mg), ascorbic acid (8000 mg), or a combination of the two reported no significant decrease in the duration of symptoms compared to standard of care [24]. This data is consistent with our results indicating that oral prophylactic supplementation of zinc through AREDS2 vitamins did not appear to protect patients from contracting COVID-19. This finding could be attributed to several factors.

The bioavailability of zinc as a therapeutic agent is highly affected by the route of administration [25]. Dietary factors and absorption are known to play a major role in the bioavailability of oral supplementation. Phytates, a compound found in whole grains, cereals, nuts, and seeds, is reported to have a chelating effect on zinc in the intestines and can cause up to almost 80% variance in zinc absorption when taken orally [26]. Furthermore, zinc uptake by enterocytes is a saturable process such that those cells that are replete with zinc typically have absorption percentages in the range of 16-50% [27]. Our study, as well as clinical trials assessing the efficacy of zinc supplementation against COVID-19, used an oral method of delivery. This may partially explain the lack of significance seen in our study, given the variability in the absorption of oral zinc and additional intestinal regulatory mechanisms.

Given the possible limited bioavailability with oral zinc supplementation, other methods of delivery have been considered, including intravenous (IV) zinc administration. This approach could result in higher bioavailability as it bypasses the major intestinal regulatory mechanisms. Furthermore, IV administration could allow for increased control of dosage. Clinical trials are currently underway in Australia to determine whether IV administration of zinc can improve clinical outcomes in patients who have contracted COVID-19 (ACTRN12620000454976). If such benefits are found, additional studies should be considered on the administration of prophylactic IV zinc supplementation. Specifically, this should be offered to those susceptible to increased morbidity and mortality due to COVID-19, including the immunocompromised and elderly population as well as those with multiple comorbidities such as chronic obstructive pulmonary diseases, obesity, cardiovascular conditions, and diabetes mellitus [28,29].

This study has several limitations. Our sample size was relatively small (110 patients who contracted COVID-19). Also, as vaccines became more prevalent, and the local numbers of positive cases decreased, the practice stopped administering the form about recent COVID-19 exposure. Therefore, the study did not examine if there were positive effects of the vaccine and zinc supplementation. The study did not examine disease severity based on zinc prophylaxis since only a few patients were hospitalized and only one mortality was encountered in the study. Given that this is a retrospective, cohort analysis, its results could be impacted by confounding variables. Patients self-reported whether they were compliant with taking their prescribed AREDS2 supplementation and, therefore, zinc intake. Although patients were thoroughly questioned regarding what vitamins they took at the time of evaluation, they may have taken additional over-the-counter zinc supplements and failed to disclose this information. Some patients may take AREDS vitamins for diagnoses other than AMD. Some patients with AMD may not take the vitamins, although patients with AMD are strongly encouraged to take the vitamins and they are queried about their compliance at nearly every visit. Also, patients who do not have AMD are encouraged not to take the vitamins since the AREDS trials did not show any benefits for such individuals. Patients with AMD tend to be older, and hence they may not travel or leave home as much as other patients, which could confound the data, albeit it would sway the data more in favor of zinc prophylaxis. We would not have been able to confirm if a patient ceased vitamin intake but still continued to report compliance. Patients may have also failed to inform us that they had contracted COVID-19. There is a possibility that asymptomatic patients existed in this study population who were unaware that they had contracted the disease. We could not account for those patients.

## Conclusions

Oral zinc supplementation has been widely purported to reduce symptomatic viral days. It has also been recommended to be used as a prophylaxis for viral infections. It has been speculated that it may help reduce the chance of contracting COVID-19. Zinc is readily available over the counter and is used in the AREDS supplements for macular degeneration. Elderly patients are at increased risk of complications from COVID-19, and hence if zinc supplementation could reduce the chance of contracting COVID-19, that would be highly beneficial. However, based on our findings, oral zinc supplementation in the form of AREDS2 vitamins is not associated with a protective effect against contracting COVID-19.

## Appendices

**Table 3:** Appendix 1. Questionnaire provided to all patients at each visit from April 1, 2020, through April 9, 2021, for assessing symptomatology and contraction of COVID-19

Name:	Date of Birth:	Medical Record Number:
Provider:		Date:
Initial screening questions:		
Do you have any of the following?	Yes	No
Cough		
Sore throat		
Congestion unrelated to seasonal allergies:		
Fever		
Nausea or vomiting		
Diarrhea		
New Loss of taste or smell		
Have you been around anyone that has tested positive for COVID-19 in the last 30 days?		
Have you tested positive for COVID-19 in the last 30 days?		

## References

[1] V'kovski P, Kratzel A, Steiner S, Stalder H, Thiel V (2021). Coronavirus biology and replication: implications for SARS-CoV-2. Nat Rev Microbiol.

[2] Feng G, Zheng KI, Yan QQ (2020). COVID-19 and liver dysfunction: current insights and emergent therapeutic strategies. J Clin Transl Hepatol.

[3] McMahon JH, Udy A, Peleg AY (2020). Remdesivir for the treatment of COVID-19 - preliminary report. N Engl J Med.

[4] Horby P, Lim WS, Emberson JR (2021). Dexamethasone in hospitalized patients with Covid-19. N Engl J Med.

[5] Rubin D, Chan-Tack K, Farley J, Sherwat A (2020). FDA approval of remdesivir - a step in the right direction. N Engl J Med.

[6] Sadoff J, Gray G, Vandebosch A (2021). Safety and efficacy of single-dose Ad26.COV2.S vaccine against Covid-19. N Engl J Med.

[7] Voysey M, Clemens SA, Madhi SA (2021). Safety and efficacy of the ChAdOx1 nCoV-19 vaccine (AZD1222) against SARS-CoV-2: an interim analysis of four randomised controlled trials in Brazil, South Africa, and the UK. Lancet.

[8] Lopez Bernal J, Andrews N, Gower C (2021). Effectiveness of Covid-19 vaccines against the B.1.617.2 (Delta) variant. N Engl J Med.

[9] Pal A, Squitti R, Picozza M (2021). Zinc and COVID-19: basis of current clinical trials. Biol Trace Elem Res.

[10] Maares M, Haase H (2016). Zinc and immunity: an essential interrelation. Arch Biochem Biophys.

[11] Maret W (2013). Zinc biochemistry: from a single zinc enzyme to a key element of life. Adv Nutr.

[12] Fukada T, Yamasaki S, Nishida K, Murakami M, Hirano T (2011). Zinc homeostasis and signaling in health and diseases: zinc signaling. J Biol Inorg Chem.

[13] Cvijanovich NZ, King JC, Flori HR, Gildengorin G, Vinks AA, Wong HR (2016). Safety and dose escalation study of intravenous zinc supplementation in pediatric critical illness. JPEN J Parenter Enteral Nutr.

[14] Hulisz D (2004). Efficacy of zinc against common cold viruses: an overview. J Am Pharm Assoc (2003).

[15] Suara RO, Crowe JE Jr (2004). Effect of zinc salts on respiratory syncytial virus replication. Antimicrob Agents Chemother.

[16] te Velthuis AJ, van den Worm SH, Sims AC, Baric RS, Snijder EJ, van Hemert MJ (2010). Zn(2+) inhibits coronavirus and arterivirus RNA polymerase activity in vitro and zinc ionophores block the replication of these viruses in cell culture. PLoS Pathog.

[17] Read SA, Obeid S, Ahlenstiel C, Ahlenstiel G (2019). The role of zinc in antiviral immunity. Adv Nutr.

[18] Gorusupudi A, Nelson K, Bernstein PS (2017). The age-related eye disease 2 study: micronutrients in the treatment of macular degeneration. Adv Nutr.

[19] McPherson SW, Keunen JE, Bird AC, Chew EY, van Kuijk FJ (2020). Investigate oral zinc as a prophylactic treatment for those at risk for COVID-19. Am J Ophthalmol.

[20] Age-Related Eye Disease Study Research Group (2001). A randomized, placebo-controlled, clinical trial of high-dose supplementation with vitamins C and E, beta carotene, and zinc for age-related macular degeneration and vision loss: AREDS report no. 8. Arch Ophthalmol.

[21] Li W, Moore MJ, Vasilieva N (2003). Angiotensin-converting enzyme 2 is a functional receptor for the SARS coronavirus. Nature.

[22] Hunter J, Arentz S, Goldenberg J (2021). Zinc for the prevention or treatment of acute viral respiratory tract infections in adults: a rapid systematic review and meta-analysis of randomised controlled trials. BMJ Open.

[23] Yao JS, Paguio JA, Dee EC (2021). The minimal effect of zinc on the survival of hospitalized patients with COVID-19: an observational study. Chest.

[24] Thomas S, Patel D, Bittel B (2021). Effect of high-dose zinc and ascorbic acid supplementation vs usual care on symptom length and reduction among ambulatory patients with SARS-CoV-2 infection: the COVID A to Z randomized clinical trial. JAMA Netw Open.

[25] Chinni V, El-Khoury J, Perera M (2021). Zinc supplementation as an adjunct therapy for COVID-19: challenges and opportunities. Br J Clin Pharmacol.

[26] Hambidge KM, Miller LV, Westcott JE, Sheng X, Krebs NF (2010). Zinc bioavailability and homeostasis. Am J Clin Nutr.

[27] Maares M, Haase H (2020). A guide to human zinc absorption: general overview and recent advances of in vitro intestinal models. Nutrients.

[28] Gold MS, Sehayek D, Gabrielli S, Zhang X, McCusker C, Ben-Shoshan M (2020). COVID-19 and comorbidities: a systematic review and meta-analysis. Postgrad Med.

[29] Wolff D, Nee S, Hickey NS, Marschollek M (2021). Risk factors for Covid-19 severity and fatality: a structured literature review. Infection.

